# Does knowledge of danger signs of pregnancy predict birth preparedness? A critique of the evidence from women admitted with pregnancy complications

**DOI:** 10.1186/1478-4505-12-60

**Published:** 2014-10-09

**Authors:** Scovia N Mbalinda, Annettee Nakimuli, Othman Kakaire, Michael O Osinde, Nelson Kakande, Dan K Kaye

**Affiliations:** Department of Nursing, School of Health Sciences, College of Health Sciences, Makerere University, P.O. Box 7072, Kampala, Uganda; Department of Obstetrics and Gynecology, School of Medicine, College of Health Sciences, Makerere University, P.O. Box 7072, Kampala, Uganda; Department of Obstetrics and Gynecology, Jinja Regional Hospital, Jinja, Uganda; Clinical, Operations and Health Services Research Program, Joint Clinical Research Centre, P. O. Box 10005, Kampala, Uganda

**Keywords:** Pregnancy, Childbirth, Knowledge about danger signs, Birth preparedness, Complication readiness, Maternal near miss

## Abstract

**Background:**

Improved knowledge of obstetric danger signs, birth preparedness practices, and readiness for emergency complications are among the strategies aimed at both enhancing utilization of maternal health services and increasing access to skilled care during childbirth, particularly for women with obstetric complications. It is unclear whether knowledge of danger signs translates into improved birth preparedness and complication readiness. The objective was to assess the association between knowledge of danger signs and birth preparedness among women admitted with pregnancy complications.

**Methods:**

The study included 810 women admitted in the antepartum period to Mulago hospital, Uganda. Data was collected on socio-demographic characteristics, reproductive history, pregnancy complications, knowledge of danger signs, and birth preparedness/complication readiness (BPCR). Logistic regression analyses were conducted to explore the relationship between knowledge of danger signs and birth preparedness.

**Results:**

Only about 1 in 3 women were able to mention at least three of the five basic components of BPCR, and could be regarded as ‘knowledgeable on BPCR’. One in every 4 women could not mention any of the five components. Women with history of obstetric problems during the previous pregnancy were more likely to be knowledgeable on danger signs when compared to those who had no complications in prior pregnancy. Women who were knowledgeable on danger signs were four times more likely to be knowledgeable on BPCR as compared to those who were not knowledgeable.

**Conclusions:**

Though awareness about danger signs was low, knowledge of danger signs was associated with knowledge of birth preparedness. More emphasis should be given to emergency/complication readiness during antenatal care sessions. There is a need to strengthen existing policy interventions to address birth preparedness and complication readiness for obstetric emergencies.

## Background

Childbirth is often associated with unpredictable life-threatening obstetric complications that lead to maternal and neonatal morbidity and mortality, especially in low-resource countries [1]. Indeed, in the absence of skilled obstetric care, 15% of all pregnant women will suffer from serious and long term morbidities and disabilities [2]. Receiving care from a skilled provider during childbirth has been identified as the most important intervention to prevent maternal and neonatal mortality [3–5]. Improved knowledge of obstetric danger signs, birth preparedness practices, and readiness for emergency complications are among strategies aimed at both enhancing utilization of maternal health services and increasing access to skilled care during childbirth, particularly for women with obstetric complications [3–6].

The Ugandan Ministry of Health launched the Safe Motherhood Healthcare package in 2004 [7], with the ultimate goal of providing obstetric interventions to reduce maternal mortality. The causes of maternal morbidity and mortality may be analyzed using the Three Delays Model [8]. This model identifies three phases of delay: delay in seeking care, delay in reaching care, and delay in receiving adequate care when reaching a health facility [8]. This model is based on the assumptions that knowledge of danger signs and preparedness for addressing obstetric complications ensures that predictable elements of the three phases of delay can be anticipated, identified in time and addressed promptly as they arise [9]. Delays in seeking care may be caused by failure to recognize signs of complications or perceive the severity of illness, cost considerations, and previous negative experiences with the healthcare system. Delays in reaching care may be caused by the long distances from a woman’s home to a health facility, poor condition of roads, and absence or unaffordability of emergency transportation. Delays in receiving care may result from negative attitudes of healthcare providers, shortages of supplies and basic equipment, a lack of healthcare personnel, and poor skills of healthcare providers.

The Maternal and Neonatal Health Program of JHPIEGO developed the birth-preparedness and complication readiness (BPCR) matrix to address these three delays at various levels to ensure that women and newborns receive appropriate, effective, and timely care [9]. These levels include the pregnant woman, her family, her community, health providers, health facilities, and policy-makers at different stages: pregnancy, childbirth, and the postpartum period. The concept of BPCR includes knowledge of danger signs, planning for a birth attendant and birth location, arranging transportation, identifying a blood donor, and saving money in case of an obstetric complication. It is unclear whether knowledge of danger signs translates into improved birth preparedness and complication readiness, yet the objective of the BPCR strategy is to promote active preparation and decision making for delivery by pregnant women and their families, as every pregnant woman faces risk of sudden and unpredictable life threatening complications. From studies in Ethiopia among pregnant women or women of reproductive age [10–12], most women were not knowledgeable about birth preparedness and complication readiness for obstetric emergencies, despite having awareness on the danger signs of pregnancy.

Studies performed in different countries have shown that health education during antenatal care enhances the utilization of skilled health care and improves mothers’ knowledge about obstetric danger signs [13–18]. During antenatal care and any other contact with pregnant women, the health education provided is supposed to raise awareness about obstetric danger signs and ensure mothers make adequate preparation for childbirth complications. Whether knowledge of danger signs translates into and is associated with improved birth preparedness has not been documented. Such an assessment would add to the evidence that specific interventions result in reduced maternal or neonatal mortality and morbidity. The objective was to explore the association between knowledge of obstetric danger signs and birth preparedness/complication readiness among women admitted in pregnancy with obstetric complications.

## Methods

### Study setting

This research was part of a mixed-methods study assessing preventable factors associated with maternal and neonatal near-miss morbidity, from the perspective of patients and healthcare providers. The study was conducted at Mulago hospital, Uganda’s national referral hospital and the teaching hospital for Makerere University. It has over 1,500 beds, of which over 400 are maternity beds, and conducts over 35,000 deliveries per year.

### Participants and data collection

Participants were women consecutively admitted to hospital from 20 to 36 weeks of gestation for complications of pregnancy and all women with pregnancy complications were eligible for inclusion into the study. The main reason for admission was febrile illness (34%). Others were anemia, hypertensive disorders, preterm labor, false labor, urinary tract infections, and anemia in pregnancy. Data was collected as an exit interview after hospital discharge using the tool on monitoring birth preparedness from JHIPIEGO [16]. This tool is used to guide assessment and monitoring of safe motherhood programs by evaluating interventions at multiple levels by identifying indicators, referred to as the BPCR Index, for each of six levels: the individual woman, her family (husband/partner), the community, the health facility, the provider, and the policymaker [19]. This tool is used to derive these indicators and in tracking progress (extent to which the indicators have been realized). The behaviors or practices identified by the tool are also labelled ‘process indicators’, because they measure processes along the pathway to maternal death or survival [20]. The tool was adapted to the local context of a hospital by eliminating the policy maker component of the instrument.

The data collected included socio-demographic characteristics such as age, marital status, level of education and occupation, number of pregnancies, number of deliveries, any abortions, number of living children, gestation age of the current pregnancy (obtained from a combination of the last normal menstrual period, the fundal height on abdominal palpation, and abdominal ultrasound examination). Other data included obstetric complications during the current and previous pregnancies. Women were asked to spontaneously cite six danger signs during pregnancy, childbirth, and immediate postpartum period, as well as two danger signs for newborns; these were open-ended questions. A woman who reported at least one danger sign in pregnancy, childbirth or postpartum period was considered to be ‘knowledgeable’ on danger signs. We also asked about awareness components of BPCR. Women who mentioned at least three of the five basic components of BPCR were regarded as ‘knowledgeable’ on BPCR.

### Data analysis

Data was entered and analyzed by using SPSS windows version 16. We compared the proportion of women who were knowledgeable about danger signs with knowledge on BPCR. The independent variables included socio-demographic characteristics, reproductive history, pregnancy complications, and being knowledgeable about danger signs (knowledge of at least one antepartum, intrapartum, and postpartum danger sign), while the independent variable was being knowledgeable on BPCR. Variables with a *P* value of <0.2 were further analyzed using the logistic regression analysis to assess factors independently associated with knowledge about BPCR.

### Ethical considerations

Ethical approval to conduct the study was obtained from the Ethics and research committees of Mulago hospital (REC 310–2012), the School of Medicine, Makerere University College of Health Sciences (REC 2012–172) and Uganda National Council for Science and Technology. Permission to conduct the study was obtained from the department of Obstetrics and Gynecology, Makerere University. All participants gave written informed consent to be interviewed.

## Results

Table 1 shows the socio-demographic characteristics. All the women had attended antenatal care at least three times prior to hospitalization. The mean age of the participants was 25.4 ± 6 years (median 25 years). The majority were of gravidity 2 to 4. Table 2 shows the knowledge on danger signs. Vaginal bleeding during pregnancy was the most commonly mentioned danger sign. Others were vaginal discharge, abdominal pain, and fever. Only 20% mentioned at least three danger signs and were regarded as knowledgeable.

**Table 1:** **Socio-demographic and obstetric characteristics of the study population**

Variables	n =810	%
**Age**		
≤25	732	90.4
>25	78	9.6
**Education level**		
Primary or no formal education	512	63.2
Secondary or higher level education	298	36.8
**Marital status**		
Single	110	13.6
Married	700	86.4
**Occupation**		
Employed	192	23.7
Not employed	618	76.3
**Gravidity**		
1	212	26.2
2–4	438	57.8
≥5	130	16
**Gestation age (wks)**		
<37	740	91.1
≥37	70	8.9
**Antenatal attendance**		
Attendance at least 4 times	535	66.0
Attended 1–2 times	245	30.2
Never attended	30	3.8
**Previous obstetric complication in this pregnancy**		
Yes	150	18.5
No	660	81.5
**Complication in a prior pregnancy (n =568)**		
Yes	218	38.4
No	350	61.6

**Table 2:** **Knowledge on obstetric danger signs, where multiple responses were elicited**

Danger sign reported	Numbers	%
*During pregnancy		
Vaginal bleeding	546	67.4
Swollen hands, face, or both	376	46.4
Blurred vision	48	5.9
Abdominal pain	62	7.7
Fever	46	5.2
Severe headache	51	6.3
*During labor and delivery		
Profuse vaginal bleeding	92	11.4
Prolonged labor longer than 12 hrs	12	1.5
Convulsions	4	0.5
Retained placenta	6	0.7
Generalized weakness or collapse	22	2.7
*Postpartum		
Severe vaginal bleeding	220	27.1
Foul smelling vaginal discharge	152	18.7
High fever	82	10.1
Abdominal pain	56	6.9
**Knowledge of three danger signs (at least at each stage)		
Yes	225	27.8
No	585	72.2

*Multiple responses.

**Knowledge of at least one danger sign in pregnancy, labor, and postpartum.

Table 3 shows the knowledge and practices on birth preparedness and complication readiness, where multiple responses were elicited. More than 2 of every 3 women identified that saving money in case of emergency or during labor is an important component of BPCR. However, only about 1 in every 4 women mentioned the need to identify means of transport, while only about 1 in 8 women mentioned the need to identify a blood donor. With these findings, only 36.5% of the respondents were regarded as knowledgeable on BPCR. While nearly 1 in every 3 women mentioned at least three components of BPCR, about 1 in every 4 women could not mention any of the five components.

**Table 3:** **Knowledge on the components of birth preparedness/complication readiness (BPCR)**

Variables	Number	Percentage (%)
*Basic component of BPCR		
Saving money for use in emergencies or during labor	544	67.2
Preparations for place of birth	194	11.6
Identifying transport in case of emergency and during labor	216	26.7
Identifying a birth companion	40	5.0
Identifying a blood donor	30	3.7
**Knowledge of three basic components of BPCR**		
Yes	256	36.5
No	514	63.5

*Multiple responses elicited.

Table 4 shows factors associated with knowledge of danger signs while Table 5 shows factors associated with knowledge of BPCR. There was a statistically significant association between not being in formal employment and lack of knowledge on BPCR (adjusted odds ratio, 0.4; 95% CI, 0.2–0.8). There was a significant association, however, between knowledge of danger signs (during pregnancy, labor and postpartum period) and BPCR (adjusted odds ratio, 3.9; 95% CI, 2.0–7.5).

**Table 4:** **Factors associated with being knowledgeable about three danger signs (n =810)**

Variables	Total n =810	Knowledgeable n =160	%	Univariate OR (95% CI)	Multivariate OR (95% CI)
**Age**					
≤25	732	14,2	19.4	1	1
>25	78	1,8	23.1	1.2 (0.5–2.7)	1.3 (0.4–3.7)
**Education level**					
Primary or no formal education	298	6,0	20.1	1	1
Secondary or higher level of education	512	1,00	19.5	1.1 (0.7–1.7)	1.9 (0.9–4.0)
**Marital status**					
Married	700	12,6	18.0	1	1
Single	110	3,4	30.9	2.0 (1.1–3.8)	0.7 (0.3–2.1)
**Occupation**					
Employed	192	4,4	22.9	1	1
Not employed	618	11,6	18.8	0.8 (0.4–1.3)	0.4 (0.2–0.8)
**Parity**					
<4	698	13,2	18.9	1	1
≥4	112	2,8	25.0	1.4 (0.7–2.7)	1.2 (0.5–2.9)
**Gestation age**					
≥37	72	1,2	16.7	1	1
<37	738	14,8	20.1	1.2 (0.5–3.2)	1.9 (0.6–6.6)
*Previous obstetric complication					
Yes	150	7,8	17.9	1	1
No	660	4,0	26.7	0.28 (0.23–0.34)	0.2 (0.14–0.41)

*Primigravida are excluded.

**Table 5:** **Factors associated with being knowledgeable on birth preparedness/complication readiness (BPCR) (n =810)**

Variables	Total n =810	Knowledgeable n =296	%	Univariate OR (95% CI)	Multivariate OR (95% CI)
**Age**					
≤25	732	266	36.3	1	1
>25	78	30	38.5	1.1 (0.6–2.2)	1.5 (0.5–4.2)
**Education level**					
Primary or no formal education	298	112	37.6	1	1
Secondary or higher level of education	512	184	35.9	0.9 (0.6–1.4)	1.3 (0.7–2.6)
**Marital status**					
Married	700	218	31.1	1	1
Single	110	78	70.9	0.23 (0.15–0.33)	0.43 (0.52–0.64)
**Occupation**					
Employed	192	90	46.9	1	1
Not employed	618	206	33.3	0.5 (0.4–0.9)	0.3 (0.2–0.6)
**Parity**					
<4	698	264	37.8	1	1
≥4	112	32	28.6	0.6 (0.3–1.2)	0.5 (0.2–1.2)
**Gestation age (wks)**					
≥36	72	36	52.8	1	1
<36	738	260	35.0	0.5 (0.2–0.9)	0.2 (0.1–0.5)
**Knowledge of danger signs during pregnancy**					
No	660	102	63.8	2.3 (2.3–2.7)	3.9 (2.0–7.5)
Yes	150	194	29.8	1	1

## Discussion

The study showed low levels of knowledge of obstetric danger signs and low levels of birth preparedness among women with pregnancy complications during the antepartum period. Only about 1 in 3 women were able to mention at least two of the five basic components of BPCR, and could be regarded as ‘knowledgeable for BPCR’. Women who were knowledgeable on danger signs were four times more likely to be knowledgeable on BPCR as compared to those who were not knowledgeable. Knowledge of at least three danger signs was independently associated with knowledge about BPCR.

The BPCR concept is based on the assumption that knowledge of danger signs leads to greater anticipation and preparation to mitigate effects of pregnancy and childbirth complications by reducing the first two delays and the third delay if health facilities are prepared to address obstetric complications. Recognition of obstetric danger signs is the key factor in seeking health care for obstetric emergencies and seeking preventive care or health promotion during pregnancy and childbirth. Therefore, a lack of awareness of obstetric danger signs is associated with a lack of preparedness for normal birth or complication readiness in case of obstetric complications that require emergency healthcare [5, 6]. With the assumption that ‘every pregnancy faces risks’, women should be made aware of danger signs of obstetric complications during pregnancy, childbirth, and the postpartum period. This empowers them to make prompt decisions for healthcare seeking in case of routine healthcare seeking or emergency complications.

The findings are in agreement with previous research [5, 7, 10–18] that shows that birth preparedness is not easy to achieve especially in developing countries, where the majority are relatively poor, few women identify transportation ahead of childbirth, few women put aside funds for transport in case of emergency, and few women are knowledgeable on danger signs or implement BPCR. This could be due to several factors [21–28]: poor antenatal care attendance, poor quality of health education during antenatal care, limited time available for antenatal care, lack of health education in hospitalized patients, rural poverty, absence of a functional referral system, and failure of the health system to implement the policy on BPCR.

Despite a limitation of being a hospital-based study, our findings raise several concerns. Firstly, while it is expected that most women hospitalized for pregnancy complications are aware of danger signs (since health education is supposed to be provided to them hospital during their hospitalization), this is not the case. The findings thus depict missed opportunities for health education during antenatal care and antepartum hospitalization. Such missed opportunities may be due to several problems such as understaffing and poor counseling (that would enable risk factor-recognition and birth preparedness) [29]. Secondly, BPCR should be made an integral part of maternal and child health services, and every opportunity should be utilized to ensure that women are informed about danger signs, the important danger signs, the implications of danger signs for safe motherhood, and what should be done to address obstetric complications. Sadly, this is not the case. Thirdly, efforts should be made to raise community awareness about birth preparedness, such as provision of emergency funds, emergency transport, and blood donors, and interventions that can be instituted at community level. Lastly, using the mass media to communicate information on danger signs might increase community awareness on birth preparedness and complication readiness for obstetric emergencies [30] through advocating for changes in attitudes, normative beliefs, and behaviors.

The implications of the findings are that interventions geared to improvement in complication readiness need to focus on raising public awareness on danger signs, and every opportunity, including hospitalization, must be utilized for health education on BPCR. Family members, particularly spouses, are keen on supporting women in developing birth plans and preparedness for birth as well as complication readiness for obstetric emergencies [18, 31, 32]. This is critical as knowledge of danger signs is a prerequisite for prompt decision-making for childbirth, emergency obstetric complications and seeking referral [33–36]. Emphasis should be given to the quality of information offered to pregnant women during antenatal care [37, 38]. While the emergency system has several components, it needs all of them to function adequately in order to maintain and sustain the ‘emergency chain of care’ [39]. Recognition that there is an emergency need is the primary step [39, 40]. The next step is community support that involves effective communication, reliable transport, adequately functioning and equipped health facilities for first aid, and community funding [39–41]. The last step is a referral facility with appropriately trained, skilled, and equipped staff who can provide prompt effective treatment and referral to a higher level facility if necessary [31]. The three components must be maintained in a state of readiness.

## Conclusions

The study shows low awareness of danger signs and birth preparedness among pregnant women admitted with pregnancy complications, which indicates a missed opportunity for health education. This makes informed decision-making problematic for pregnant women and healthcare providers. However, women who were knowledgeable about danger signs were more knowledgeable about BPCR. New strategies are needed to inform pregnant women about BPCR so as to improve decision-making in pregnancy and childbirth. This calls for urgent need to utilize all available opportunities to raise awareness of obstetric danger signs and to strengthen health education and counseling on BPCR.

## Abbreviations

BPCR: Birth-preparedness and complication readiness.
